# Health Tract, No. 136

**Published:** 1865

**Authors:** 


### HEALTH TRACT, No. 136.

### HOUSEHOLD VERMIN,

INCLUDING rats, ants, cockroaches, bed-bugs, body-lice, etc. These are to citizens what weeds are to farmers, compelling all to work for a living; and work gives a good appetite, a vigorous digestion, sound sleep, general health, and a good old age. It may be a question of ethics, whether we ought to set our wits to work in devising any short cuts in the direction of exterminating the house pests above named. Until our doctors of divinity settle this point, the safer side may be taken of erring from ignorance, rather than overt design, if it be an error to wage a war of extermination against every living thing which occupies your premises without your consent, and without paying for " board and lodging."

Prevention is the safest and noblest remedy; of these, personal and habitational cleanliness and a big tom-cat are perfectly efficient. But the number of clean housekeepers in the city of New York is not over one in a hundred, judging from the gangrenous green which defaces the "risers" in the steps which lead into our brown-stone mansions, and the unswept condition of the gutter part of the street-way, in front of most dwellings. And if any of our readers are curious to see sights, let them " happen in" at some of the " auctions of household furniture," which are so numerous in any April in New-York; auctions in first-class houses of families " going to the country," " breaking up housekeeping," or " going to Europe," meaning three times out of four, perhaps, a " financial smash-up." Let any reader go into any dozen such places, and judge for himself as to the supply of good housekeepers, tidy and clean, in this great Gotham. But do not judge from the condition of the parlors and parlor furniture, but look into cellars and sinks, and closets and attics; inspect bed-ticks and mattresses, and " comfortables" and woolen blankets. Such sights! And then again, what loads of abominations in the cellar! What piles of bones and bottles; of old shoes and wads of fat; pork-skins, fish-heads, empty mackerel-kits, and Scotch herring-boxes; and other things, too numerous and suggestive to mention! So that if tidiness were the only remedy for house-vermin, New-York would soon be like Egypt in olden time, when noisome insects swarmed on the food as it was being passed into the mouth.

BODY VERMIN breath through their sides; common sweet oil plugs up their air conduits, and death from suffocation is speedy and certain, always. Ignorance in many cases makes the oil, which is the efficient remedy, merely the vehicle for applying a poison dangerous to man, which has no efficiency whatever in destroying vermin.

ROACHES devour greedily, and die while eating, flour paste, if into half a pint of it, while hot, a dime's worth of phosphorus is stirred, in a tin cup, with a long stick. When this is nearly cold, a quarter as much grease, to keep it from drying; then smear it on broken glass or dirty board, to be left where they congregate.

THE PERSIAN POWDER is harmless to man, but certain death to insects. It is the powdered blossoms and flowers of a Caucasian vegetable, called " Pyrethrum Roseum," of a yellowish gray, odorless, tasteless at first, but leaving a burning sensation. The plant will flourish in our country, and seeds will be furnished by the Agricultural Department at Washington City. Address Hon. J. Newton, Department of Agriculture, Washington, D. C. It is the best remedy known, because cheap, perfectly harmless to man, and infallibly fatal to insects.

HOUSE-FLIES.—Take as much each of ground black pepper and sugar as will lie on a dime, moisten with two teaspoons of cream or rich milk, and spread it on a plate or board; the flies eat it, seek the air, and die out of doors. Or mix the liquor of boiled poke-root with a little molasses, and spread it about on plates.

The powder of Coculus Indicus, which boys use to stupefy fishes, destroys many insects, if scattered about their haunts.

As for rats, it is best to keep a good cat or terrier-dog; or keep every thing eatable on shelves hanging from the ceiling or around the walls. Chloride of lime, wrapped in a rag and stuffed in rat-holes or passage-ways, will sometimes drive them from the house for a few months, until the chlorine odor has disappeared. Five cents' worth of strychnine, mixed in three table-spoons of corn-meal, with a few drops of anise, attracts the rats, but it is too dangerous a substance to come into any household. A table-spoon of plaster-of-Paris in powder, mixed with a pint of Indian meal, with grated cheese or oil of anise, is safe and effectual. Ten grains of powdered phosphorus, mixed with a pint of Indian meal, is a good remedy. Powdered potash, strewn in their paths, makes their feet sore, and drives them away. Rats are too cunning to be caught long by any kind of trap. But there is nothing so efficient as a good-mannered, well-trained cat; dogs annoy neighbors by their barking.

